# Antioxidant Saffron and Central Retinal Function in ABCA4-Related Stargardt Macular Dystrophy

**DOI:** 10.3390/nu11102461

**Published:** 2019-10-15

**Authors:** Marco Piccardi, Antonello Fadda, Francesco Martelli, Dario Marangoni, Adriano Magli, Angelo M. Minnella, Matteo Bertelli, Stefano Di Marco, Silvia Bisti, Benedetto Falsini

**Affiliations:** 1 Dipartimento di Scienze dell’Invecchiamento, Neurologiche, Ortopediche e della Testa-Collo, Fondazione Policlinico Universitario A. Gemelli- IRCCS, 00168 Rome, Italy; piccmarc@tiscali.it (M.P.); dariomarangoni80@yahoo.it (D.M.); aminnella59@gmail.com (A.M.M.); 2 Dipartimento di Malattie Cardiovascolari, endocrino-metaboliche e invecchiamento, Istituto Superiore di Sanità, 00168 Rome, Italy; afadda@iss.it (A.F.); francesco.martelli@iss.it (F.M.); 3 Department of Ophthalmology, Scheie Eye Institute, University of Pennsylvania, Philadelphia, PA 19104, USA; 4 Dipartimento di Oftalmologia Pediatrica, Università di Salerno, 84084 Salerno, Italy; amagli@unisa.it; 5 Istituto di Oftalmologia, Università Cattolica del S. Cuore, 00168 Rome, Italy; 6 Laboratorio di Genetica Medica e Biologia Molecolare, MAGI, 38068 Rovereto, Italy; matteo.bertelli@assomagi.org; 7 Dipartimento di Scienze Cliniche Applicate e Biotecnologie, Università dell’Aquila, 67100 L’Aquila, Italy; stefano.dimarco@univaq.it (S.D.M.); s.bisti@team.it (S.B.); 8 Istituto Nazionale Biosistemi e Biostrutture, 00168 Rome, Italy; 9 Istituto Italiano di Tecnologia, NetS3 Laboratory, Neuroscience and Brain Technologies (NBT), 16100 Genova, Italy

**Keywords:** ABCA4 gene mutation, antioxidants, focal electroretinogram, neuroprotection, photoreceptors, saffron, retinal function, personalized medicine

## Abstract

Retinal oxidative damage, associated with an ATP-binding cassette, sub-family A, member 4, also known as ABCA4 gene mutation, has been implicated as a major underlying mechanism for Stargardt disease/fundus flavimaculatus (STG/FF). Recent findings indicate that saffron carotenoid constituents crocins and crocetin may counteract retinal oxidative damage, inflammation and protect retinal cells from apoptosis. This pilot study aimed to evaluate central retinal function following saffron supplementation in STG/FF patients carrying ABCA4 mutations. Methods: in a randomized, double-blind, placebo-controlled study (clinicaltrials.gov: NCT01278277), 31 patients with ABCA4-related STG/FF and a visual acuity >0.25 were randomly assigned to assume oral saffron (20 mg) or placebo over a six month period and then reverted to P or S for a further six month period. Full ophthalmic examinations, as well as central 18° focal electroretinogram (fERG) recordings, were performed at baseline and after six months of either saffron or placebo. The fERG fundamental harmonic component was isolated by Fourier analysis. Main outcome measures were fERG amplitude (in µV) and phase (in degrees). The secondary outcome measure was visual acuity. Results: supplement was well tolerated by all patients throughout follow-up. After saffron, fERG amplitude was unchanged; after placebo, amplitude tended to decrease from baseline (mean change: −0.18 log µV, *p* < 0.05). Reverting the treatments, amplitude did not change significantly. fERG phase and visual acuity were unchanged throughout follow-up. Conclusions: short-term saffron supplementation was well tolerated and had no detrimental effects on the electroretinographic responses of the central retina and visual acuity. The current findings warrant further long-term clinical trials to assess the efficacy of saffron supplementation in slowing down the progression of central retinal dysfunction in ABCA4-related STG/FF.

## 1. Introduction

Stargardt disease (STG) is the most common hereditary recessive macular dystrophy [1] characterized by juvenile to young adult onset, central visual impairment, progressive bilateral atrophy of the macula and retinal pigment epithelium (RPE), with a frequent appearance of orange/yellow flecks distributed around the macula and/or the mid retinal periphery [2]. A clinically similar retinal disorder, fundus flavimaculatus (FF), often displays later ages of onset and slower progression. It has been suggested and demonstrated [3] that STG and FF represent allelic disorders. Mutations in the gene encoding an ATP-binding cassette (ABC) transporter (ABCR), mapping to chromosome 1p13-p21, have been found to be responsible for STG/FF [3]. The ABCR gene is expressed at high levels in the retina, in both rod and cone photoreceptors [4]. Weng et al., (1999) [5], investigating the molecular mechanisms underlying photoreceptor degeneration in ABCR knock-out mice, proposed that photoreceptors die as a consequence of RPE ’poisoning’ by lipofuscin accumulation and loss of RPE supportive role. Accumulation within the RPE cells of a compound, A2E, forming from the condensation of phosphatydilethanlolamine (PE) and the all-trans-retinal released from photoactivated rhodopsin [6], probably leads in vivo to increased absorption of blue lights and phototoxic RPE cell damage. The mutation-induced disease may affect both rod and cone photoreceptors, at relatively early stages. In vitro studies [7] also demonstrated that the ABCR itself is an efficient target of all-trans-retinal-mediated photo-oxidative damage.

The vulnerability of photoreceptors to the oxidative mechanism and their protection by antioxidant lutein and zeaxanthin has been demonstrated in the past (see, for example, Kim et al., 2006 [8]). Recent experimental findings [9] indicate that Saffron, derived from the pistils of *Crocus sativus*, may exert protection against oxidative damage. Saffron has been shown to be protective, for both morphology and function, in a rat model of light-induced photoreceptor degeneration [9]. In this model, cell death is thought to result from oxidative stress induced by a prolonged increase in oxygen tension and photo-oxidation. Saffron is a promising candidate to be tested because the stigmata of *Crocus sativus* contain biologically high concentrations of the carotenoids crocins and crocetin [10], whose multiple C=C bonds give the antioxidant potential. Besides, Crocins can activate metabolic pathways to protect cells from apoptosis and to reduce light-induced death in isolated photoreceptors [11,12], while crocetin [10] increases oxygen diffusivity through liquids, such as plasma. Kanakis et al., (2007) [13] showed that metabolites of antioxidant flavonoids bind directly to DNA and induce its partial conformation to beta-DNA, thereby protecting the cell from damage. Microarray studies [14] showed that saffron pre-treatment, while protecting the rat retina from light damage, up-or-down-regulated a significant number of known genes that are involved in the retinal response to light exposure. Some of these genes, like that of endothelin 2 and fibroblastic growth factor might have a direct effect also on retinal function. The peculiar characteristics of saffron constituents suggest involvement of very different ways of action going from antioxidant activity to gene expression direct control. These components may act in humans as protective agents against oxidative damage. Indeed, the results of an early pilot trial in patients with early age-related maculopathy support this effect [15]. These data have been recently confirmed from three independent groups [16,17,18].

The goal of this study was to evaluate, in a randomized, placebo-controlled trial (clinicaltrials.gov: NCT01278277), central retinal function following short-term saffron supplementation in STG/FF patients with an ABCA4 genotype. The rationale was that saffron carotenoid constituents, acting as protective agents against oxidative damage, may counteract photoreceptor dysfunction/loss associated with STG/FF. In this pilot study, the macular cone-mediated electroretinogram (ERG) in response to high-frequency flicker (focal ERG, FERG), a sensitive index of central retinal function [19,20,21], was assessed and monitored as the main outcome variable.

## 2. Materials and Methods 

The research followed the tenets of the Declaration of Helsinki. Written, informed consent was obtained from each patient before the inclusion in the study and after the aims and procedures of the research were fully explained. The study was approved by the Ethics Committee/Institutional Review Board of the Catholic University.

Patients: a group of 31 STG/FF patients (14 males, 17 females) with an established ABCA4 genotype, accumulated prospectively over an interval of 12 months at the outpatient service of the Institution, were included in this study. Patients met the following inclusion criteria: 

- Macular and peripheral retinal degeneration with typical funduscopic lesions (retinal flecks) and a cone-rod pattern of retinal dysfunction, as determined by standard Ganzfeld electroretinography and dark-adapted fundus perimetry, 

- Relatively preserved central retinal function (visual field by Goldmann V/4e > 30°, corrected EDTRS visual acuity >0.25) and stable fixation as determined by a Visuskope, 

- Two causative mutations in ABCA4 gene, or one causative mutation and one or more of the following clinical findings: a dark choroid sign, documented pisciform flecks, macular atrophy, reduced visual acuity or central scotoma on Humphrey 10-2 visual field testing, 

- At least four follow-up clinical examinations over the past three years, 

- No or minimal ocular media opacities, 

- No concomitant ocular (e.g. glaucoma, amblyopia) or systemic diseases. 

Demographic, clinical and molecular data of patients are reported in Table 1. None of the patients was taking medications (e.g. chloroquine) that are known to affect the macular function or interfere with carotenoid absorption. Table 1 reports detailed demographic, clinical and molecular genetic data for each patient included in the study.

Mutation screening was performed by single-strand conformation polymorphism (SSCP) strategy of the whole coding region of ABCA4 [22]. Direct sequencing was also performed on siblings of probands and parents, when available, to confirm segregation of alleles.

For a detailed characterization of phenotype, all patients underwent before inclusion full assessment of their fundus lesions, by Cirrus OCT and near-infrared autofluorescence imaging. Functional assessment was also completed by automated static perimetry (Humphrey 10–2) and in some cases by Nidek microperimetry. However, none of these fundus or functional parameters were included among the outcome variables, given the short-term duration of this pilot study. Twenty-five out of 31 patients had a deterioration of their visual acuity (>one line) or fERG parameters over the three years preceding the inclusion in the study. The remaining five patients had reported visual acuity deterioration over the preceding five years.

### 2.1. Treatment and Testing Schedule 

The saffron used in these trials has been provided by Hortus Novus Srl. and corresponds to Saffron Repron (Trademark N°.86895965), having chemical characterization described in the patent N° W02015/145316 filed on 20 March 2015, owned by Hortus Novus Srl.

The patients were divided into two groups: 14 were treated with oral supplementation of a daily dose of saffron (S) for 180 days, and 17 assumed oral placebo (P) during the same period. At the end of the 180 days, patients were crossed-over and, and after a one-week wash-out period of, assigned respectively to P or S supplementation. The assumption supported also by the results of a previous clinical trial [15], was that the effect of S supplementation rapidly vanishes once interrupted. In all patients, clinical examination, including visual acuity testing with a calibrated standard Snellen chart and fundus examination by direct and indirect ophthalmoscopy, and fERG testing, were performed at the study entry (baseline) and after 180 days of S or P. In all cases, compliance was judged by telephone interview and pill counts. Major and minor adverse events or any medication side effects were reported.

### 2.2. Electrophysiological Methods 

fERG testing was performed according to a previously published technique [19]. Briefly, ERGs were elicited by the LED-generated sinusoidal luminance modulation of a circular uniform field (dominant wavelength: 660 nm, 18° in diameter, 80 cd/m2 mean luminance, 93.5% modulation depth), presented at the frequency of 41 Hz on the rear of a Ganzfeld bowl uniformly illuminated at the same mean luminance as the stimulus. fERGs were recorded monocularly using Ag-AgCl superficial cup electrodes taped over the skin of the lower eyelid. A similar electrode, placed over the eyelid of the contralateral, patched eye, was used as reference (interocular ERG^25^). FERG signals were amplified, band-pass filtered between 1 and 250 Hz (−6 dB/oct), sampled with 12-bit resolution, (2 kHz sampling rate), and averaged. A total of 2400 events (in 12 blocks of 200 events each) were averaged. The sweep duration was kept equal to the stimulus period. Single sweeps exceeding a threshold voltage (25 μV) were rejected to minimize the noise coming from blinking or eye movements. A discrete Fourier analysis was performed off-line to isolate the fERG fundamental harmonic, whose amplitude (in μV) and phase (in degrees) were estimated. Component amplitude and phase were also calculated separately for partial blocks (200-events packets) of the total average, from which the standard error (SE) of amplitude and phase estimates were derived to test response reliability. Averaging and Fourier analysis were also performed on signals sampled asynchronously at 1.1 times the temporal frequency of the stimulus, to give an estimate of the background noise at the fundamental component. For each response, a signal-to-noise ratio (S/N) was calculated as the ratio between the peak-to-peak amplitude of the fundamental harmonic and the peak-to-peak noise amplitude, estimated at the same harmonic component. The S/N value provides, in principle, a more accurate measure of the true fERG signal, especially when it is intrinsically low and more easily influenced by noise, as in STG/FF patients. The calculated S/N was expressed in dB units (1 = 0.05 log10 units).

In all subjects, the fERG testing protocol was started after a pre-adaptation period of 20 min to the stimulus mean illuminance. Pupils were pharmacologically (Tropicamide 1%) dilated to 8–9 mm. Subjects fixated (from a distance of 30 cm) at the center of the stimulation field with the aid of a small (15 min of arc) fixation mark.

### 2.3. Statistical Analysis 

Main outcome variables were fERG amplitude (in µV) and phase (in degrees). The secondary outcome was best-corrected visual acuity.

For each patient included in the study, the averaged data from both eyes were included as a single entry in the statistical analysis. In all analyses, SEs and 95% confidence intervals of the means were considered for between-group comparisons. FERG amplitude data underwent logarithmic transformation to better approximate normal distribution.

Sample size estimation of patients for this study were based on previous investigations [23,24], where the between- and within-subjects variability (expressed as data SD) of fERG parameters was determined in normal subjects and STG/FF patients. Assuming between- and within-subjects SDs in fERG amplitude and phase of 0.1 log micro V and 20 degrees, respectively, the sample sizes of patients assigned to both S and P provided a power of 80%, at an alpha = 0.05, for detecting in each group a test-retest difference (i.e. 180 days minus baseline test) of 0.1 log micro V (SD : 0.1) and 30 degrees (SD : 20) in amplitude and phase, respectively. Given the absolute mean amplitude and phase values of the patients’ fERG, these differences were considered as clinically meaningful, since they corresponded approximately to a 25–30% change in either amplitude or phase. The patients’ sample size also provided a power of 90%, at an alpha = 0.05, for detecting within-group differences in fERG amplitude and phase changes (i.e. 180 days minus baseline test) of 0.15 log micro V (SD : 0.1) and 40 degrees (SD : 20), respectively.

Electrophysiological results were analyzed by repeated-measures analysis of variance, ANOVA, with multiple predefined contrasts. Dependent variables in the ANOVA design were fERG log amplitude, S/N and phase. Treatment (S vs P) was the independent variable. Visual acuity changes across treatments were analyzed either individually for every patient or as group means by repeated-measures ANOVA, assuming a normal distribution. 

In all the analyses a *p* < 0.05 was considered as statistically significant.

## 3. Results

In all patients enrolled in the study, the study supplement and placebo were well tolerated and free of any major or minor adverse effect. Compliance was judged to be optimal with >99% of pills assumed by all patients during the follow-up period. 

Electrophysiological data of each patient, recorded at baseline, after the first and the second treatment period are reported in Table 2.

**Figure 1:**
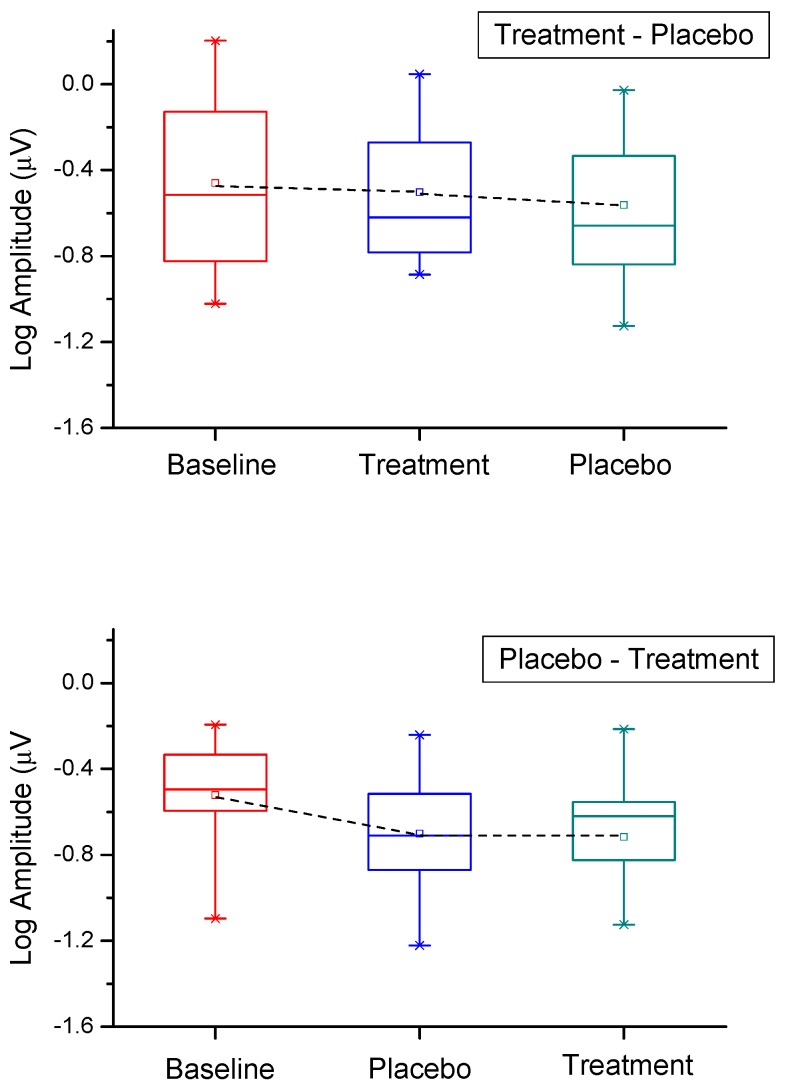
Box plots the fERG amplitude values recorded at baseline and after six months of S (Treatment) or P (Placebo) supplementation, following the cross-over study design. The symbol and line in the middle of the box indicate the mean and the median, the box lower and upper boundaries the 25th and 75th percentiles, and the lower and upper whiskers the 5th and 95th percentiles, respectively.

Figure 1 shows as box plots the fERG amplitude values recorded at baseline and after six months of S (Treatment) or P (Placebo) supplementation, according to the cross-over study design. The symbol and line in the middle of the box indicate the mean and the median, the box lower and upper boundaries the 25th and 75th percentiles, and the lower and upper whiskers the 5th and 95th percentiles, respectively. It can be noted that, in the group of patients (*n* = 14) starting the trial with S, there was at the end of this period only a minimal, non-significant, reduction in fERG amplitude, compared to baseline. After patients starting with S were switched to P, fERG amplitude at the end of this period also tended to decrease slightly and non-significantly (*p* ns), compared to either baseline or to the S time point. In the group of patients (*n* = 17) starting the trial with P, there was at the end of this period a more substantial and significant reduction in fERG amplitude (mean 0.18 log units, standard error, SE 0.04), compared to baseline values. After patients starting with P were switched to S, no changes in fERG amplitude were found at the end of this period, compared to the preceding S time point, indicating a stability of the response amplitude. In patients starting with S, repeated-measures ANOVA did not show any significant change in mean fERG amplitude (F (2,28): 1.63, *p* ns) throughout the follow-up period. In patients starting with P, ANOVA showed a significant change across follow-up times (F (2,15) 4.2, *p* = 0.02) due to the loss in fERG amplitude from baseline following P supplementation.

**Figure 2:**
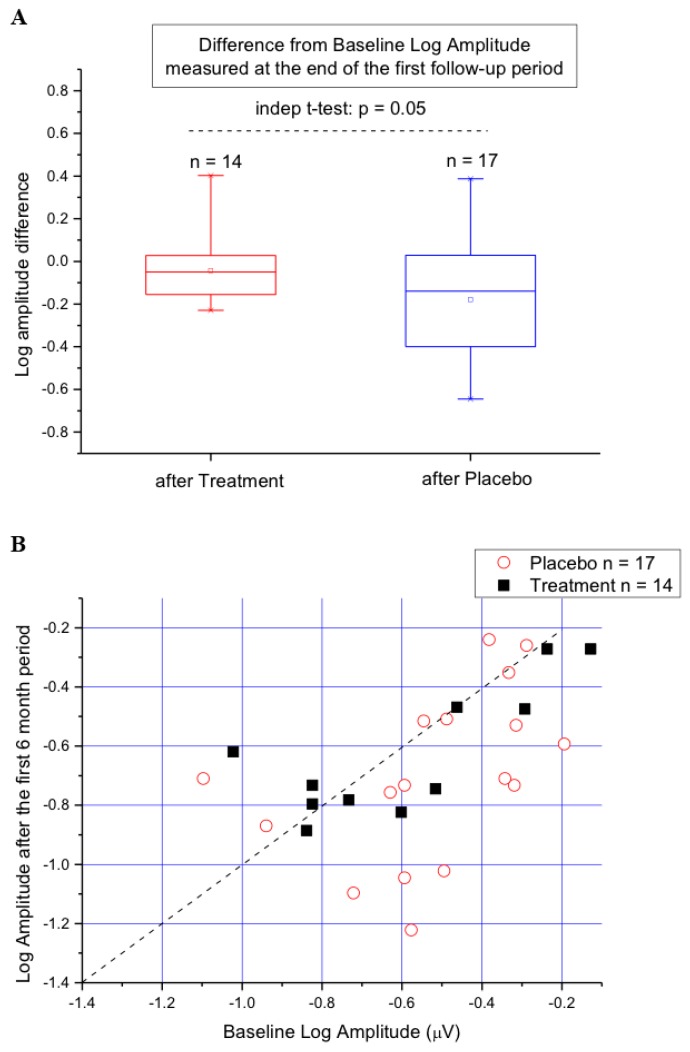
(**A**) Box plot showing the difference from baseline fERG Log amplitude recorded at the end of the first six-month study period in patients assuming S (Treatment) and those assuming P (Placebo). (**B.**) The same changes recorded in A shown as a scatter of data points. The diagonal line in the plot indicates equivalence. It can be noted that in seven out of 17 patients assuming P, fERG amplitude losses were substantially larger compared to those recorded in the 14 patients assuming S.

The changes in fERG amplitude from baseline values recorded after the first six months of S (Treatment) or P (Placebo) supplementation are evaluated in more detail in Figure 2A,B. In the box plot of Figure 2A, the difference from baseline Log amplitude measured at the end of the first period is compared between patients assuming S (Treatment) and those assuming P (Placebo). It can be noted that this difference tended to be more negative in the latter compared to the former patients. The between-group difference shown in the Figure approached statistical significance (*p* = 0.058). In Figure 2B, the same changes recorded in A are shown as a scattering of data points. The diagonal line in the plot indicates equivalence. Data falling to the right of the line indicate amplitude loss from baseline. It can be noted that in seven out of 17 patients assuming P, fERG amplitude losses from baseline were of a magnitude that was never found in any of the 14 patients assuming S.

fERG phase and visual acuity did not show any significant change throughout the study period.

Long-term (36 months) clinical and electrophysiological follow-up was obtained, outside the clinical trial period, in 22 out of the 31 patients enrolled in the study. All these patients continued saffron treatment in an open-label fashion. Follow-up was every 8–12 months. Although not part of the original study design, the results were evaluated to check for the long-term safety of the supplementation and to determine the feasibility of a future long-term study. Fifteen out of 22 patients who assumed saffron for an additional 36 months, retained the visual acuity they had at enrollment. Seven patients lost 2 lines of visual acuity. fERG amplitudes and phase did not change significantly, on average, in patients with stable visual acuity, while tended to decline, on average, in patients with acuity loss. Fundus imaging autofluorescence showed a tendency to increase of the central hypo-autofluorescent area in all 22 patients. In all patients evaluated in the long-term follow-up, no side effects of saffron supplementation were recorded.

## 4. Discussion

The present pilot study was designed to evaluate central retinal function following oral supplementation of a *Crocus sativus* extract S, in patients suffering from ABCA4-related STG/FF retinal dystrophy. The results showed that six months of S supplementation was well tolerated and had no detrimental effects on the electroretinographic responses of the central retina. The findings also suggest that S supplementation may prevent progressive deterioration of macular fERG amplitude, as observed in patients assigned first to P and then to S treatment, although the overall amplitude data did not show unequivocally an S treatment effect. In recording small ERG signals from the central retina, a critical aspect is to assess the noise influence on the response [19,25,26,27,28] and to determine how the uncorrelated noise affects the true signal amplitude estimate. In the past, we developed a steady-state fERG technique allowing a real-time estimate of the noise level at a frequency close to that of interest [15,19,20,23,27,29,30]. According to our method, and as previously published [19,23], the S/N ratio at the fundamental harmonic provides a reliable estimate of the true response amplitude, which may be employed, for instance, to assess retinal threshold to flicker modulation in the macular region [19]. In the present study, noise assessment was instrumental for increasing the precision of our estimate of the fERG signal, given the unfavorable condition of very low amplitude, typical of patients with STG/FF dystrophy.

In the present study, we did not measure retinal autofluorescence (the clinical biomarker of lipofuscin) in the patient retinas for both technical reasons and reduced study duration. Experiments on the ABCA4 knock-out mice are now in progress and seems to suggest that saffron treatment induces a reduction in lipofuscin accumulation.

To our knowledge, this is the first randomized, double-blind, placebo-controlled study evaluating the safety and efficacy of a potential antioxidant treatment in ABCA4-related retinal degenerations. A previous non-randomized lutein treatment study in patients with ABCA4-related retinal degenerations, without placebo control, was reported by Aleman et al., 2007 [31]. The authors showed that lutein supplementation increased the levels of macular pigment optical density, without changing significantly their foveal-macular sensitivity. Interestingly, untreated patients with ABCA4 mutations showed a loss of macular pigment optical density, compared to age-matched controls, implying that ABCA4 dysfunction is somewhat linked to the production and efficiency of macular pigment carotenoids.

It has been recently shown that crocins and crocetins are derivatives of macular carotenoid zeaxanthin [32]. It could be hypothesized that an abnormality in the ABCA4 gene may produce a loss, quantitative or qualitative, of the carotenoid zeaxanthin. This could lead to a local loss of crocins and crocetins, to reduced protection of macular photoreceptors, and ultimately to an enhanced apoptotic photoreceptor death as a consequence of A2-PE mediated oxidative damage [8]. The exogenous supplementation of crocins and crocetin, made available in the S extract, may correct, at least in part, this condition and counteract the effect of oxidative damage to macular photoreceptors. After saffron oral supplement, crocins are hydrolyzed in the intestinal tract and rapidly absorbed as crocetin and subsequently eliminated (max 8 hrs) from human plasma [33]. Saffron metabolites have been searched in AMD patients (blood and urine) and in animal models with induced retinal degeneration (stressed retina, blood, urine, kidney, liver, and cerebral cortex) and relative controls [34]. It was possible to quantify metabolites in healthy control while in patients apparently metabolites were immediately absorbed and used. Results obtained in animal suggested a re-synthesis of crocins in stressed retinas. Even though no metabolites search was performed in the present clinical trial in which we may hypothesize a similar pattern. It is important to point out that metabolites do not accumulate in blood and urine and apparently reach only stressed eyes and undergo chemical modifications. Moreover, recent studies demonstrated that saffron is a multitask drug able to mitigate retinal neurodegeneration by acting at different levels: reducing ATP-induced retinal cytotoxicity by targeting P2X7 receptors, interacting with the endocannabinoid system and with extracellular matrix [35,36,37]. The macular fERG is an index of photoreceptor/bipolar cell function in the macula [19,23,27]. This index has proven to be highly sensitive and reliable in detecting loss of photoreceptor function and its progression in retinal degenerations [20,21]. It is important to note that short-term S supplementation, although unable to ameliorate in a statistically significant way fERG response, clearly demonstrated a tendency in the stabilization of retinal function. fERG remained stable when patients started with S by contrast, when the P patients were reverted to S supplementation, fERG amplitude tended to stabilize.

This study did not evaluate the long-term effect of S supplementation. STG/FF retinal degeneration may be rapid in the central retina, but relatively slow in the pericentral retinal region and retinal periphery [38]. The main clinical outcome parameter in this study was the macular fERG, which is known to reflect the function of outer/middle retina in the central visual field but cannot be directly compared with the currently employed clinical parameters of visual function, such as visual acuity and central field sensitivity. It is worth noting that visual acuity, a secondary outcome parameter, was unchanged in this trial. Although visual acuity has proven to be correlated with fERG amplitude in many clinical studies, it may reflect the function of the foveal region, which represents only a small fraction of the central 18° of retina area, whose integrated function is sampled by the fERG. Therefore, it may well be that the changes observed in the fERG may not have been reflected in the visual acuity of patients. Clinically, many reports have documented an abnormal functioning of both macular and peripheral cones, as well as rods, in STG/FF [39,40,41]. Characteristic abnormalities of rod dark adaptation [42] and the recovery of cone sensitivity after bleaching [24], have been reported. Further studies would be needed to address the long-term effect of S supplementation on retina-wide function, psychophysical macular sensitivity and dark adaptation, and the relationship of treatment effect with the ABCA4 genotype.

Long-term follow-up of 36 months was obtained outside the period of the clinical trial in a subgroup of our patients. These data cannot be part of the statistical assessment of the saffron supplementation, but they suggest a long-term safety of supplementation and possible visual stabilization. No clear indications have been obtained about the efficacy of saffron treatment that should be tested in future studies with appropriate placebo control.

## 5. Conclusions

In conclusion, short-term S supplementation was well tolerated and had no detrimental effects on the electroretinographic responses of the central retina. The current findings warrant further long term trials evaluating major clinical parameters (visual acuity, central perimetric sensitivity, fundus autofluorescence,) as the main outcome measures, to assess the efficacy of S supplementation in slowing down the progression of central retinal dysfunction in ABCA4-related STG/FF, especially in childhood, given that it is non-invasive, well-tolerated and free of adverse effects.

## Figures and Tables

**Table 1:** Demographic, Clinical and Molecular Data of Patients. Patient ID number (#)

Patient # Sex	Acuity		Baseline	1st CONTR	2nd CONTR	Mutation I Allele	Mutation II Allele
	OD	OS					
*2. F, 14*	0.2	0.2	B 11.04.11	A 24.10.11	18.06.12	IVS35 + 2t > c	IVS40 + 5g > a
*3. M, 11*	0.1	0.1	B 11.04.11	A 10.10.11	16.04.12	c432A > G;	Q767d > a G1961e
*4. F, 35*	0.1	0.1	A 11.04.11	B 11.10.11	16.04.12	c.52C > T; p.Arg18Trp	C7671 > A; p.Val767Asp
*5. F, 14*	0.3	0.3	A 18.04.11	B 24.10.11	23.04.12	c.1622T > C; p.Leu541Pro	------
*6. M, 12*	0.3	0.3	A 18.04.11	B 24.10.11	23.04.12	c.1622T > C; p.Leu541Pro	------
*8. M, 56*	0.1	0.5	B 18.04.11	A 24.10.11	18.05.12	Arg653His	Arg653His
*9. M, 61*	0.9	0.9	B 18.04.11	A 24.10.11	18.05.12	Arg653His	Arg653His
*11. M, 12*	0.2	0.2	A 02.05.11	B 25.11.11	08.05.12	c.5882G > A; p.Gly1961glu	c.6764G > T, p.Ser2255Ile
*12. F, 16*	0.2	0.2	B 09.05.11	A 08.11.11	22.05.12	c.3602T > G; p.Leu1201Arg	c.5530G > T; Gly1844Cys c.5722G > T; p.Glu1908X
*14. F, 78*	0.1	0.1	B 16.05.11	A 28.11.11	21.05.12	c.4793C > AAla1598Asp	(c.6184_6188delGTCT; p.Val2062fsX2113)
*18. F, 34*	0.8	0.8	B 20.06.11	A 19.12.12	21.06.12	c.2099G > A; p. W700X	c.4561C > T; P1486L
*19. F, 47*	1.0	1.0	A 11.07.11	B 09.01.12	09.07.121	c.2690C > T; p.Thr897Ile	------
*20. M, 43*	0.1	0.1	A 11.07.11	B 09.01.12	09.07.12	c.2690C > T; p.Thr897Ile	------
*21. M, 12*	0.1	0.1	B 11.07.11	A 09.01.12	09.07.12	c.4139C > T; Pro1380Leu	c.6005+1G > C
*23. M, 34*	0.1	0.1	B 20.06.11	A 19.12.11	25.06.12	c.61C > T; p.Gln21Ter	c.5882G > A; p.Gly1961Glu
*24. M, 16*	0.1	0.9	A 12.10.11	B 23.04.12	29.10.12	c.2461T > A; p.Trp821Arg c.2459A > G; p.Tyr850Cys	c.5882G > A; p.Gly1961Glu
*25. M, 29*	0.1	0.1	A 07.11.11	B 02.05.12	12.11.12	C768G > T; p.Val256splice	c.4437G>A;p.Trp1479X
*26. M, 32*	0.1	0.1	B 07.11.11	A 28.05.12	03.12.12	5714 + 5G-A	6088C > T
*27. F, 29*	0.2	0.2	A 08.11.11	B 04.06.12	17.01.13	Thr977Pro	IVS40 + 5G-A
*28. M, 27*	0.2	0.2	B 21.11.11	A 21.05.12	22.11.12	c.5065T > C; p.S1689P	c.5882G > A; p.G1961E
*29. F, 22*	0.1	0.5	A 13.12.11	B 18.06.12	20.12.12	4709-4711delA	Gly1961Glu
*30. M, 42*	0.1	0.2	A 23.01.12	B 27.06.12	07.01.13	R152Q	R653C
*31. M, 10*	0.2	0.2	B 13.02.12	A 06.08.12	11.02.13	c.4200C>A;p.Tyr1400X	c.686T>C;p.Leu229Pro
*32. M, 17*	0.3	0.3	A 20.02.12	B 03.09.12	18.03.13	R18W	C1490Y
*33. M, 22*	0.4	0.4	B 21.02.12	A 10.09.12	09.03.13	c.5882G>A;p.Gly1691Glu	(c.3994_4017dup;p,.Gln1332_cys1339dup) Val2062ThrfsX52
*38. M, 15*	0.2	0.2	B 03.04.12	A 15.10.12	22.04.13	c.2791G>A;p.Val931Met	------
*39. M, 21*	0.1	0.2	A 04.04.12	B 29.10.12	13.05.13	Ser1064Pro	IVS35+2t>c
*42. F, 21*	0.3	0.3	B 17.04.12	A 08.10.12	11.04.13	Ivs13+1g>a	Ivs40+5g>40
*43. M, 52*	0.1	0.5	B 23.04.12	A 12.11.12	16.05.13	c.3323G>A;p.Arg1018His	c.5882G>A;p.Gly1961Glu
*44. M, 35*	0.4	0.4	A 17.05.12	B 13.12.12	20.06.13	GlY1961Glu	IVS45+1g>c
*45. F, 56*	0.8	0.1	B 21.5.12	A 05.11.12	02.05.13	c.2549>G;P.Tyr850Cys c.2875A>G;p.Thr959Ala	c.5882G>A;p.Gly1961Glu

**Table 2:** Electrophysiological Data of Patients. Data not classified (n.c.)

	Baseline	SN1	Noise I	1st period	SN1	Noise I	2nd Period	SN1	Noise I
Pat. ID	*Amp (ph)* *OD; OS*	*OD* *OS*	*OD* *OS*	*Amp (ph)* *OD; OS*	*OD* *OS*	*OD* *OS*	*Amp (ph)* *OD; OS*	*OD* *OS*	*OD* *OS*
*2.*	0.63: 0.30 −160.5; −69.0	15.70 9.26	0.10 0.10	0.40; 0.49 −5.8; −177.3	0.86 6.12	0.17 0.27	0.24; 0.28 −67.3; 148.0	8.16 18.55	0.09 0.03
*3.*	0.14; 0.50 −51.1; 10.7	−11.61 10.93	0.54 0.13	0.12; 0.07 −108.4; 146.1	−4.23 −10.51	0.20 10.23	0.09; 0.28 152.1; −86.6	−1.05 4.43	0.10 0.17
*4.*	0.33; 0.35 23.6; 163.0	6.30 3.09	0.16 0.25	0.13; 0.55 10.6; 44.6	13.49 11.21	0.03 n.c.	0.39; 0.05 −32.1; 20.5	7.19 −8.92	0.17 0.13
*5.*	0.97;0.71 27.1; −161.4	4.41 n.c.	0.29 n.c.	0.70; 0.63 19.6; −132.9	21.56 14.17	0.06 0.12	0.84; 0.84 48.0; −164.6	13.32 14.73	0.18 0.15
*6.*	0.8; 0.858 2.0; −146.0	9.92 n.c.	0.28 n.c.	0.90; 0.92 42.1; −129.1	22.73 14.98	0.06 0.11	0.32; 0.61 42.2; 150.6	12.78 14.98	0.07 0.11
*8.*	0.34; 0.17 −53.1; 30.2	9.04 1.70	0.12 0.10	0.12; 0.06 −179.2; 113.2	−1.00 8.18	0.04 0.02	0.09; 0.08 −103.6; 70.9	10.57 2.64	0.01 0.04
*9.*	0.63; 0.65 −54.1; −173.3	9.73 4.05	0.21 0.41	0.28; 0.23 −80.5; −175.8	6.79 −1.39	0.13 0.27	0.04; 0.11 −153.5; 115.0	−11.34 −2.65	0.13 0.14
*11.*	0.79; 0.70 −173.0; 18.5	14.86 10.57	0.14 0.21	0.75; 0.32 12.6; −177.8	10.47 4.35	0.23 0.19	0.40; 0.33 22.4; −142.2	10.70 10.07	0.12 0.10
*12.*	0.18; 0.33 −139.3; 116.7	−0.04 7.10	0.18 0.15	0.19; 0.18 99.1; −36.5	9.00 9.54	0.07 0.06	0.20; 0.38 −124.2; −124.5	19.63 12.61	0.02 0.09
*14.*	0.24; 0.33 140.0; 176.5	4.53 2.75	0.14 0.24	0.41; 0.20 −89.7; −42.6	1.12 −0.76	n.c. n.c.	0.37; 0.40 145.2; −133.5	17.16 0.77	0.05 0.37
*18.*	0.10; 0.28 −33.7; 167.4	−5.02 17.98	0.11 0.03	0.13; 0.03 −40.1; 135.2	7.83 −5.87	0.05 0.07	0.11; 0.19 −47.9; −0.9	7.02 17.08	0.05 0.03
*19.*	1.33; 1.86 −3.3; −174.4	n.c. 20.35	n.c. 0.18	1.22; 1.01 −9.5; −159.8	21.99 16.39	0.07 0.15	0.70; 0.48 110.4; −81.0	23.78 18.27	0.05 0.06
*20.*	0.1; 0.089 3.6; 56.9	2.93 9.98	0.13 0.25	0.32; 0.16 −20.3; 19.5	11.61 −5.72	0.08 0.30	0.20; 0.07 −36.5; −25.6	4.49 −6.04	0.12 0.14
*21.*	0.37; 0.46 −9.4; −173.6	11.85 11.55	0.10 0.12	0.89; 0.26 −15.3; −144.8	10.01 13.00	0.28 0.06	0.05; 0.12 −47.9; 127.6	5.25 0.20	0.01 0.05
*23.*	0.50; 0.53 41.6; −141.6	9.34 22.92	0.17 0.04	0.57; 0.53 −11.7; −177.4	7.33 16.68	0.25 0.08	0.23; 0.28 −60.1; 137.7	10.32 16.82	0.07 0.04
*24.*	0.10; 0.20 −55.4; 163.3	1.50 7.72	0.08 0.08	0.24; −9.0 0.13; −141.1	6.45 6.69	0.12 0.06	0.21; 0.05 −30.6; 85.1	23.02 −3.01	0.01 0.07
*25.*	0.14; 0.16 −121.4; 146.4	−0.02 −2.6	0.14 0.10	0.15; 0.17 26.7; −80.2	4.94 6.98	0.09 0.07	0.09; 0.06 29.8; 64.9	1.92 −4.92	0.08 0.10
*26.*	0.32; 0.21 12.5; 84.4	2.93 −0.36	0.23 0.22	0.10; 0.02 −12.3; −53.3	−536 −13.28	0.18 0.11	0.04; 0.11 −159.6; −90.5	−8.29 5.79	0.11 0.06
*27.*	0.46; 0.15 48.7; −124.1	6.60 1.69	0.23 0.12	0.16; 0.20 −43.7; 142.4	10.77 −3.07	0.05 0.06	0.34; 0.04 −0.4; 42.1	8.41 0.46	0.13 0.04
*28.*	0.59; 0.37 35.3; −120.8	18.07 22.20	0.07 0.03	0.29; 0.08 −171.2; −12.6	14.59 4.81	0.05 0.05	0.31; 0.18 −53.3; 104.6	10.66 11.73	0.10 0.05
*29.*	0.57; 0.45 32.9; −155.2	12.33 3.98	0.14 0.28	0.49; 0.18 −46.8; 167.1	18.43 3.78	0.06 0.12	0.19; 0.18 16.4; 70.6	2.43 −3.11	0.15 0.26
*30.*	0.22; 0.07 49.4; 172.2	11.65 7.71	0.06 0.03	0.15; 0.11 −63.9; 148.0	16.07 7.63	0.02 0.04	0.22; 0.07 49.4; 172.2	11.65 7.71	0.06 0.03
*31.*	0.45; 0.46 34.7; −126.2	13.83 12.98	0.09 0.12	0.21; 0.18 −35.7; 165.8	10.43 14.03	0.06 0.04	0.29; 0.27 −18.2; 170.6	9.68 10.01	0.10 0.08
*32.*	0.30; 0.20 −42.0; 135.5	9.26 −0.96	0.10 0.22	0.18; 0.12 −95.9; 85.9	10.72 4.90	0.05 0.07	0.38; 0.33 −92.2; 118.0	7.84 4.85	0.16 0.19
*33.*	0.07; 0.16 −3.22; −9.3	0.60 4.32	0.06 0.10	0.20; 0.07 −17.4; −87.7	12.79 2.59	0.05 0.05	021; 0.10 −61.8; 136.7	4.76 7.40	0.12 0.04
*38.*	0.57; 0.40 −17.4; 137.2	14 n.c.	12 n.c.	0.35; 0.24 −20.0; −149.0	12.18 11.13	0.09 0.07	0.65; 0.57 −76; −84	26.77 11.13	0.03 0.15
*39.*	0.20; 0.17 −145.8; −68.8	−3.99 6.96	0.32 0.08	0.16; 0.17 47.0; 67.5	6.41 0.94	0.08 0.15	0.44; 0.35 −36.1;−22.2	14.02 16.3	0.09 0.06
*42.*	0.07; 0.09 34.7; −47.9	2.73 2.38	0.04 0.07	0.21; 0.18 −20.1; 127.0	4.14 8.12	0.09 0.07	0.27; 0.21 −48.9; −157.9	3.89 3.67	0.17 0.14
*43.*	0.34; 0.27 −71; −140	12.50 n.c.	0.08 n.c.	0.32; 0.30 −2.7; −29	4.31 12.69	0.20 0.07	0.17; 0.41 −41.7; −28.5	15 20.5	0.03 0.04
*44.*	0.49; 0.46 −157; −142	10.31 30.1	0.08 0.01	0.16; 0.28 −87.6; −6.6	20.66 6.83	0.01 0.10	0.39; 0.30 −20.9; −10.6	4.31 11.11	n.c. 0.11
*45.*	0.16; 0.31 13.4; −168.1	11.13 17.47	0.04 0.08	0.27; 0.08 −27.9; −84.6	21.91 −4.51	0.02 −0.13	0.17; 0.18 −80.9; −57	25.21 11.43	0.01 0.06

## Abbreviations

STG/FF	Stargardt disease/fundus flavimaculatus
S	Saffron
P	Placebo
ERG	electroretinogram
fERG	focal electroretinogram
FERG	high-frequency flicker ERG
RPE	retinal pigment epithelium
FF	fundus flavimaculatus (FF)
PE	phosphatidylethanolamine
ETDRS	Early Treatment Diabetic Retinopathy Study chart
SSCP	single-strand conformation polymorphism
